# Validation of an Effective Protocol for *Culicoides* Latreille (Diptera: Ceratopogonidae) Detection Using eDNA Metabarcoding

**DOI:** 10.3390/insects12050401

**Published:** 2021-04-30

**Authors:** Yoamel Milián-García, Lauren A. A. Janke, Robert G. Young, Aruna Ambagala, Robert H. Hanner

**Affiliations:** 1 Department of Integrative Biology, University of Guelph, 50 Stone Rd E, Guelph, ON N1G 2W1, Canada; laurenashleyann@hotmail.ca (L.A.A.J.); ryoung04@uoguelph.ca (R.G.Y.); rhanner@uoguelph.ca (R.H.H.); 2 Canadian Food Inspection Agency/Government of Canada, Winnipeg, MB R3E 3M4, Canada; aruna.ambagala@canada.ca

**Keywords:** eDNA metabarcoding, *Culicoides*, biosurveillance, morphology, molecular identification

## Abstract

**Simple Summary:**

All organisms shed genetic material into the environment, which is known as environmental DNA. Current molecular technologies allow for sequencing molecular markers in complex environmental samples. The use of these methods permits an effective identification and monitoring of flighted insects such as *Culicoides* species. These biting midges are agricultural pests of significant economic concern. This study identified *Culicoides* species using a novel molecular-based approach for this group and compared these results to morphological identifications of the specimens collected. There were forty-two *Culicoides* specimens collected in total, using a saturated salt solution as a collection fluid. Molecular identification detected four species. Using morphological identification, we identified two out of these four taxonomic ranks at the species level and one at the subgenus level. The inconsistency in identifying *Culicoides* specimens to the species level indicates the need for curated DNA reference libraries for molecular-based identification. The saturated salt solution used in the traps preserved the morphological characteristics and the organisms’ environmental DNA, which is an essential contribution of this study.

**Abstract:**

eDNA metabarcoding is an effective molecular-based identification method for the biosurveillance of flighted insects. An eDNA surveillance approach maintains specimens for secondary morphological identification useful for regulatory applications. This study identified *Culicoides* species using eDNA metabarcoding and compared these results to morphological identifications of trapped specimens. Insects were collected using ultraviolet (UV) lighted fan traps containing a saturated salt (NaCl) solution from two locations in Guelph, Ontario, Canada. There were forty-two *Culicoides* specimens collected in total. Molecular identification detected four species, *C. biguttatus*, *C. stellifer*, *C. obsoletus,* and *C. mulrennani.* Using morphological identification, two out of these four taxonomic ranks were confirmed at the species level (*C. biguttatus* and *C. stellifer*) and one was confirmed at the subgenus level (*Avaritia* [*C. obsoletus*]). No molecular detection of *Culicoides* species occurred in traps with an abundance of less than three individuals per taxon. The inconsistency in identifying *Culicoides* specimens to the species level punctuates the need for curated DNA reference libraries for *Culicoides*. In conclusion, the saturated salt (NaCl) solution preserved the *Culicoides’* morphological characteristics and the eDNA.

## 1. Introduction

Environmental DNA (eDNA) metabarcoding is a powerful tool in biomonitoring and biosurveillance [1,2]. It allows for the identification of multiple species from complex environmental samples by taking advantage of high-throughput sequencing (HTS) technologies and already established DNA reference databases [1,3,4,5,6]. eDNA metabarcoding has proven to be an efficient barcode-based identification method for the biosurveillance of native and invasive insect pests [2]. This approach can include the detection of insect vectors of zoonotic diseases, which are often difficult to detect using conventional surveying methods [2,5]. The higher detection rate is particularly beneficial when implementing measures to mitigate environmental threats. The early detection of vectors and potential invasive species can be important for eradicating or containing the negative impacts often occurring after the introduction of a non-native species. The time between the introduction, establishment, or spread of a pest and the detectability of its environmental impact is critical. When the damage caused by a pest is measurable by conventional methods, the number of pest species is excessively higher than can be identified by only risk assessment [2].

The genus *Culicoides* Latreille (Diptera: Ceratopogonidae) is a group of biting midges, including known agricultural pests of significant economic concern [7,8]. *Culicoides* are called ‘no-see-ums’ or punkies, and many of their distinguishing morphological features can only be examined using a microscope due to their small size [9]. This genus comprises of 1347 known extant species globally [7], 150 of which are found in the Nearctic [10]. There are currently 38 known species of *Culicoides* in Ontario, Canada (Table S1). Traditional taxonomy and the identification of *Culicoides* rely on adult morphological characteristics [11], and it is therefore difficult to identify *Culicoides* at the other life stages of egg, larva, and pupa. Even adult *Culicoides* specimens can be challenging to identify due to their small size, making DNA barcode-based identification methods a useful tool for this genus. Further to this problem is the difficulty in accessing and utilizing published taxonomic keys for *Culicoides*, and the high taxonomic expertise required to use them.

Species in this genus are vectors of 66 viruses, 15 protozoa, and 26 species of nematodes [12]. These vectored pathogens include important livestock viruses such as the bluetongue (BTV) and Schmallenberg viruses and pathogens causing epizootic hemorrhagic disease and African horse sickness [11]. *Culicoides sonorensis* is a known vector of BTV, with a historical range throughout western North America, with only recent identifications in Ontario [13]. To monitor the range shift of *C. sonorensis*, and other *Culicoides* species that vector diseases of concern, a biomonitoring program using an eDNA metabarcoding approach for early detection while retaining morphological specimens is needed.

Traditional sampling methods for collecting *Culicoides* specimens include ultraviolet (UV) light traps that have either water containing drops of unscented liquid soap, ethylene glycol, or propylene as a collection fluid [14,15]. Using water with soap can result in specimen rot before being transferred to either ethanol or isopropanol for long-term storage. Glycol-based collection fluids are expensive and recognized as poisonous, limiting their safe use in public spaces. Alcohol-based fluids for transporting specimens are also disadvantageous due to their flammability, and therefore require special handling and transportation restrictions [16,17].

Using a salt solution as a substitute collection fluid allows for short-term specimen preservation during field placement and transportation to facilities for long-term storage processing. The use of inexpensive saturated salt (NaCl) water solutions has already proven useful for preserving arthropod morphological structures and as a reservoir of eDNA [16,17]. Using a saturated salt solution can eliminate the need for alcohol-based substances as a field collection fluid and removes the requirements of hazardous materials permits for shipping. Consequently, collection, specimen transportation, and even immediate specimen identification can occur without the need to transfer the collected specimens from a saturated salt solution to alcohol-based fluids. It saves time, reduces manipulation, and thereby reduces the risk of contamination. Sample manipulation increases the risk of physically destroying the delicate specimens or affecting key morphological taxonomic characteristics of soft-bodied organisms, so reducing the handling helps to reduce the risk of these errors from occurring. 

eDNA metabarcoding salt trap collection protocols are also advantageous as they allow for the species detection of trapped organisms, regardless of their physical integrity. They also allow for bulk sample identification containing entire organisms, or eDNA from multiple organisms, which involves the capacity to detect multiple insect pests and vectors in other species’ presence. They permit rapid and cost-effective barcode-based identification, saving the time-consuming effort of sorting individuals, which is a step needed for other molecular-based identification tools such as DNA barcoding. Besides, curated DNA reference libraries with taxonomic classification information associated with them facilitate molecular identification even in the absence of taxonomic expertise. These reference databases, such as the Barcode of Life Data System (BOLD, http://www.boldsystems.org/), will act as permanent repositories of taxonomic information that can be accessed as needed [18]. Consequently, the reference databases’ taxonomic data can then be linked to the sequence matches obtained by HTS [2]. In the present study, we validate for the first time an eDNA metabarcoding protocol for the molecular identification of *Culicoides* spp. using a saturated salt trap solution as a collection fluid. We also assess the differences between eDNA-based identification methods and traditional morphology-based methods to detect *Culicoides* spp. in Ontario, Canada.

## 2. Materials and Methods

### 2.1. Sampling

**Figure 1:**
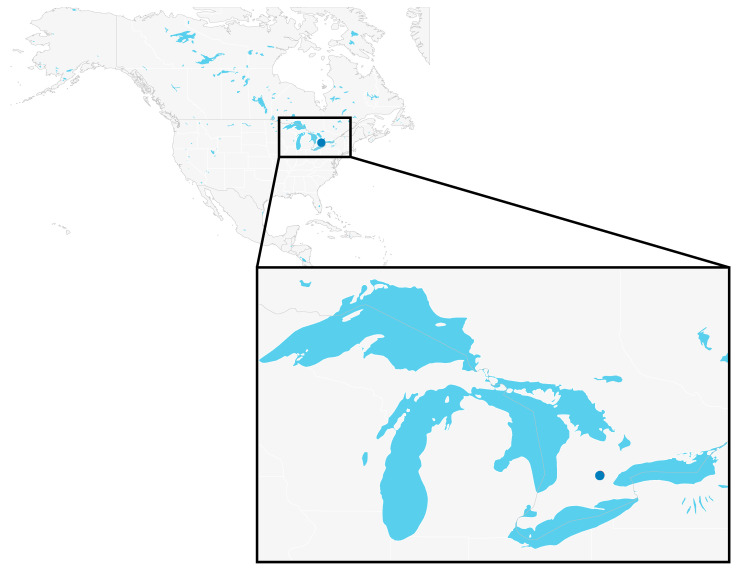
Map of North America showing collection sites (dots). A link to an interactive version of the map is also provided (Supplementary Material S1).

Miniature CDC (Centre for Disease Control) light traps with UV lights (Bioquip, CA, USA) were used to collect *Culicoides* specimens in the field. Traps were filled with a saturated salt solution prepared by mixing 1 L of water, 0.4 kg of NaCl, and two dish soap drops to break the surface tension of the collection fluid. Two traps were placed on the University of Guelph campus in Guelph, Ontario, Canada: one in the Arboretum (hereafter referred to as Arboretum trap) in a short shrubbery near a small stream (43°32′17.4624″, −80°13′6.4086″), and one in the Ontario Veterinary College Dairy Barn (hereafter referred to as Dairy Barn trap) within the cattle enclosure (43°31’43.305″, −80°13′50.1738″) (Figure 1).

Each trap collection basin was filled ¾ full with saturated salt solution, where two drops of unscented dish soap was added to break surface tension, and set out on 19 July 2019 in the late afternoon around 3 p.m. The traps were both collected the next morning at around 8:30 a.m. Once in the lab, the bulk insect samples were immediately filtered out from the salt trap solution using a sifter and put into 95% ethanol for long-term storage at −20 °C. The trap collection fluid was stored in DNA clean whirl-pak bags at −80 °C until filtration.

### 2.2. Morphological Identification

**Figure 2:**
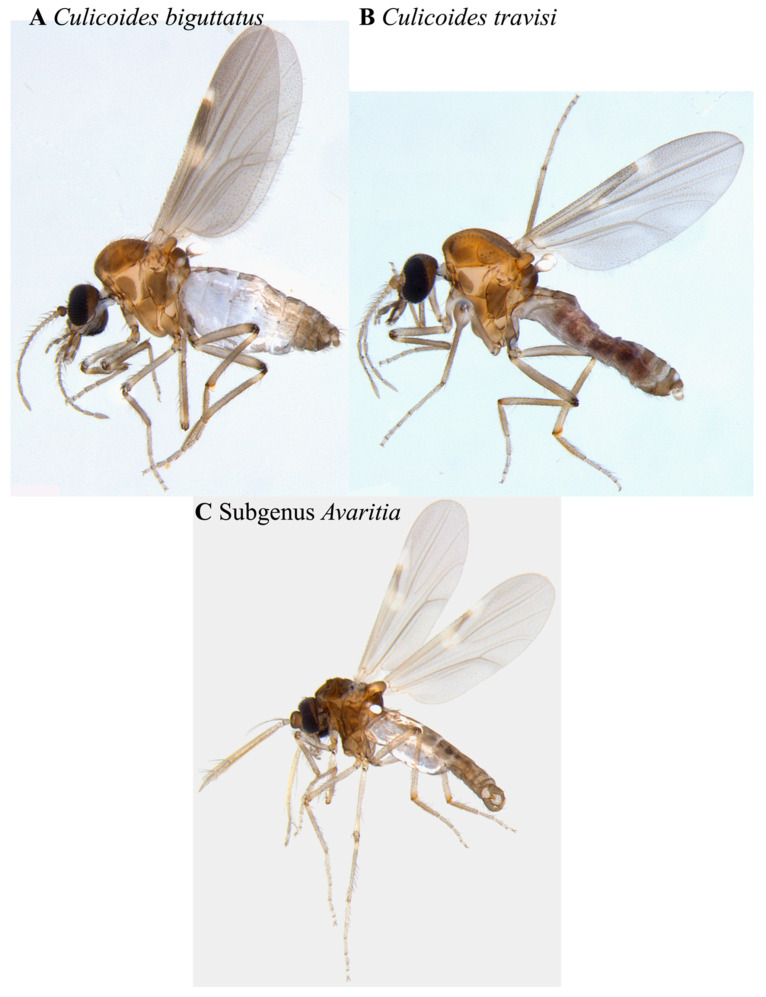
Representative images of *Culicoides* spp., morphologically identified from the Arboretum trap, showing a left lateral orientation. Specimens (**A**,**B**) are female, and specimen (**C**) is male.

*Culicoides* were sorted from the other specimens collected in the traps using a Leica stereomicroscope and a generic key from the Manual of Nearctic Diptera Vol. 1 for Ceratopogonidae (Diptera) [9]. Each specimen was imaged using the program Leica LAS X (version 3.6.0.20104; Leica Microsystems, Wetzlar, Germany), the camera Leica MC170 HD, and the scope M205 A (Leica Microsystems, Wetzlar, Germany). Images for six orientations (right lateral, left lateral, ventral, head, genitalia, and wing) were taken, and each specimen was housed individually in a 96-well microplate in 95% ethanol. These *Culicoides* samples were keyed out to the species level [19,20,21,22,23] using both the physical specimens and the high-quality images. We did not identify the ten *Culicoides* specimens in the subgenus *Avaritia* Fox down to species because we could not clearly define the distinctive features on either the specimens or the images. However, the specimens themselves remained intact for the duration of identification (Figure 2).

### 2.3. Salt Trap Solution Filtration

After removing intact specimens, the remaining saturated salt (NaCl) solutions were filtered through a nitrocellulose mixed ester membrane filter (pore size 1 µm, diameter 47mm, Sterlitech) to capture eDNA. The filter was mounted onto a magnetic filtration cup (Pall) held by a 3-port manifold connected to a GAST vacuum pump (GAST Manufactured, Inc., Benton Harbor, MI, USA). Sterilization of all supplies was conducted using 50% bleach or ELIMINase (Decon Labs). Following filtration, the membranes were stored at −80 °C until DNA extraction. 

### 2.4. DNA Extraction

All molecular analyses were performed in the Hanner laboratory at the University of Guelph, Ontario, Canada and using the protocols described in Milián-García et al., 2020 [16]. A modified CTAB buffer [24,25] (2% *w*/*v* cetyltrimethyl ammonium bromide, 2% *w*/*v* polyvinylpyrrolidone, 1.4M NaCl, 100 mM Tris-HCL, 20 nM EDTA) was used for tissue and cellular lysis due to recurrent success retrieving quality eDNA yield in the literature [26,27,28,29]. Each filter was cut into quarters using sterile razor blades, and each quarter was placed into individual 2 mL microcentrifuge tubes containing ~250 mg of 1mm-diameter glass beads. After adding 500 μL of CTAB buffer pre-warmed to 65 °C, the tubes were then placed in a TissueLyser II (Qiagen) and agitated at a frequency of 30.0 Hertz for one minute. The tubes were then incubated at 65 °C for one hour. Following the addition of 500 μL of chloroform–isoamyl alcohol, all tubes were briefly vortexed and then centrifuged at 13,000× *g* for 15 min to enable phase separation. The upper DNA-containing aqueous phase (approximately 500 μL) was transferred to new 2.0 mL centrifuge tubes. The aqueous phase was then mixed with 500 μL of isopropanol and 200 μL of 5M NaCl solution.

After a brief vortex, the tubes were stored at −20 °C overnight to facilitate DNA precipitation. DNA was then pelleted through centrifugation at 13,000× *g* for 15 min. After careful disposal of the supernatant, the pellet was washed with 200 μL of 70% ethanol. The ethanol was decanted and replaced anew after centrifugation at 13,000× *g* for 15 min. The washing step was repeated, followed by brief vortexing and centrifugation at 13,000× *g* for 15 min. Once the ethanol had been decanted for a final time, the remaining ethanol was removed. The tubes were then placed in the fume hood to allow the residual ethanol to evaporate (approximately 45 min). After a visual inspection indicated no remaining ethanol, the DNA pellet was suspended in 25 μL 1X TE buffer (pre-warmed to 70 °C). DNA extracts from each filter quarter obtained from the same trap were pooled in 1.5 mL LoBind tubes (Eppendorf). DNA’s presence and quality were evaluated using 1% agarose gel electrophoresis, and the DNA was quantified (ng μL^−1^) by fluorometry (Qubit). All DNA extracts were normalized (5 ng/μL) to ensure the same starting DNA concentration per sample in further sample processing. All DNA extracts not consumed in this experiment were placed in long-term storage at −20 °C.

### 2.5. Library Preparation

A high-throughput sequencing library for the COI gene was prepared in two PCRs [16,30]. First, a ~407 base-pair segment of the mitochondrial genome (mtDNA) was amplified as a single fragment for all samples with the addition of Illumina adaptors. The first PCR was conducted in a thermal cycler (Eppendorf Mastercycler) using the forward primers mLepF1_MiSeq/RonMWASPdeg_MiSeq and the reverse primers LepR1_MiSeq/HCO2198_MiSeq [16]. Each reaction was adjusted to 25 μL final volume, containing 2.5 μL of DNA template, 12.5 μL of 2X KAPA HiFi HotStart Ready Mix (Roche Diagnostics), and 0.2 μM of each primer. PCR cycling conditions were as follows: 94 °C (2 min), followed by 5 cycles of 94 °C (40 s), 45 °C (40 s), 72 °C (1 min), then, 35 cycles of 94 °C (40 s), 51 °C (40 s), 72 °C (1 min), and a final extension at 72 °C (5 min) [31]. One negative control was included during the amplification. The PCR products were checked on 1% agarose gel, and then purified using a 0.8× NGS magnetic beads (Machery-Nagel) ratio and following the manufacturer’s protocols.

Using de novo synthesized index primers equivalent to the Nextera XT Index Kit (Genomics Facility at the Advance Analysis Centre of the University of Guelph), a second PCR incorporated dual indices sets to the COI–Illumina adaptor sequence amplicons. The second PCR was conducted in 50 μL reaction volumes on an Eppendorf Mastercycler thermal cycler, adding unique index primer combinations for each sample. Each reaction contained 25 μL of 2× KAPA HiFi HotStart Ready Mix (Roche Diagnostics), 10 μL of molecular biology grade water, 5 μL of each index primer (10 μM), and 5 μL from the first cleaned up PCR product. The PCR profile was as follows: 95 °C (3 min), 8 cycles of 95 °C (30 s), 55 °C (30 s), 72 °C (30 s), and a final extension of 72 °C (5 min) [16,30]. Before sequencing, PCR products were visualized on 1% agarose gel and purified using a 0.6× NGS magnetic beads (Machery-Nagel) ratio and following the manufacturer’s protocols. The second PCRs were conducted in different rooms and PCR workstations than the first PCR, a standard setup for eDNA library preparation. DNA extracts and post-PCR products were treated and processed in separated areas to minimize contamination. 

### 2.6. High-Throughput Sequencing

Sequencing was performed at the Genomics Facility of the Advanced Analysis Centre (AAC) at the University of Guelph. The sequencing libraries were normalized using the SequalPrep Normalization Kit (Thermo Fisher Scientific, Waltham, MS, USA), pooled, quantified with the Qubit dsDNA HS assay kit (Thermo Fisher Scientific), and checked for fragment size in a Bioanalyzer HS DNA Chip (Agilent). Libraries passing the quality control were sequenced on an Illumina MiSeq System using a MiSeq reagent kit v3 (600 cycles), keeping 1% of the run per sample. Demultiplexing and adapter trimming of the sequencing reads were conducted with the MiSeq Reporter software, generating two paired-end raw FASTQ files.

### 2.7. Data Analysis

Quality control of each raw data file (FASTQ files) was completed using FastQC (http://www.bioinformatics.babraham.ac.uk/projects/fastqc/). COI data analysis was conducted in mBRAVE (multiplex barcode research and visualization environment). mBRAVE (http://www.mbrave.net/) is a multi-user platform supporting the storage, validation, and visualization of HTS data. The parameters in the mBRAVE platform were set as follows: (1) trimming (trim front: 30 bp; trim end: 30 bp; trim length 450 bp); (2) filtering (min QV: 20 qv; min length: 200 bp; max bases with low QV [˂20]: 25%; max bases with ultra-low QV [˂10]: 5%); (3) pre-clustering threshold: none; ID distance threshold: 3%; minimum OTU size: 5; OTU threshold: 2%); (4) assembler min overlap: 20 bp, assembler max subs: 5 bp; (5) Resulting sequences from this protocol were queried against libraries assembled from BOLD records, and matched to a BIN when possible. A BIN is an alphanumeric code representing a molecular operational taxonomic unit within the BOLD platform. Each BIN represents a putative species and will accompany taxonomic information present for the records included in the BIN [32]. 

An independent pipeline for data analysis was applied to the FASTQ raw data files to confirm the occurrence of *Culicoides* spp. in the traps. The FASTQ files were sequentially analyzed using Geneious Prime version 2021.1.1 (Biomatters, Ltd., Auckland, New Zealand) as follows: (1) paired reads were set; (2) sequences were trimmed with BBDuk version 38.84, keeping a minimum sequence quality of 20 Phred (i.e., 99% base call accuracy), and a minimum length of 200 bp; (3) paired-end reads were merged with BBMerge version 38.84; (4) duplicates were removed with Dedupe 38.84; (5) clustering was done by de novo assembly using Geneious Prime version 2020.2.2 assembler, as well as a minimum overlap identity of 98%; (6) the nucleotide basic local alignment search tool (BLASTn) was used to compare our consensus sequences library against the GenBank database of the National Center for Biotechnology Information (NCBI).

## 3. Results

### 3.1. Morphological Identification

**Figure 3:**
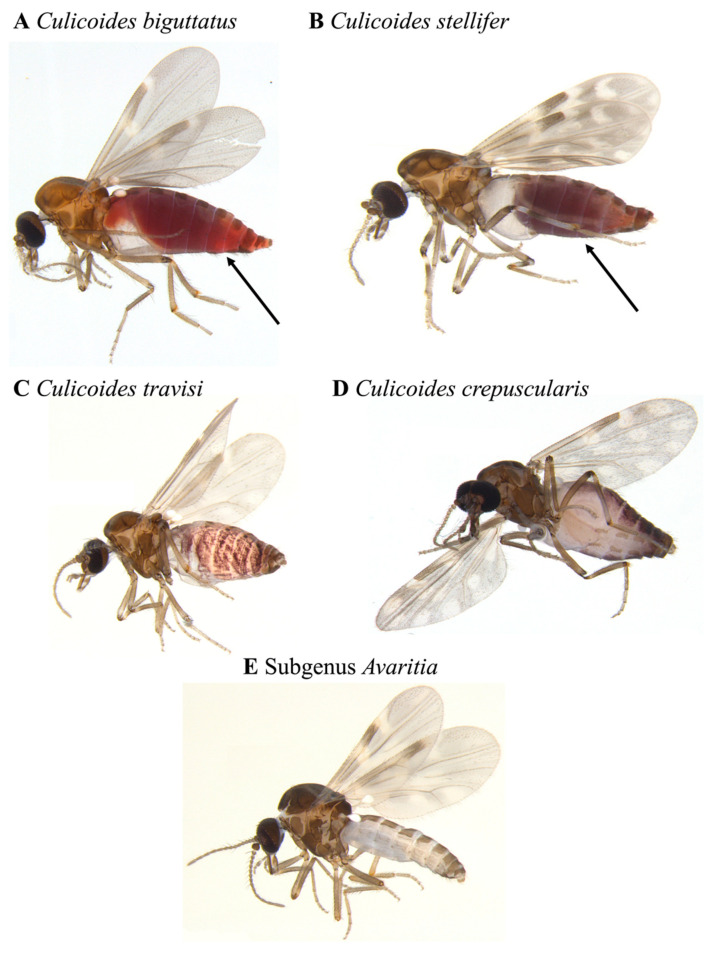
Representative images of *Culicoides* specimens, morphologically identified from the Dairy Barn trap, showing a left lateral orientation. Arrows indicate abdomens containing a blood meal. Specimens (**A**–**E**) are females.

In the Arboretum trap, we found a total of seven *Culicoides* specimens from three taxonomic groupings (Figure 2). Two out of the three taxonomic groupings were identified to the species level, and one was identified to the subgenus level (*Avaritia*). The abundance of each taxonomic group ranged from one to three (Table 1). In the Dairy Barn trap, a total of 35 *Culicoides* specimens were collected belonging to four species, with the remainder belonging to the subgenus *Avaritia* and not identified to the species level (Figure 3). The abundance per taxonomic grouping in this trap varied from 1 to 13 (Table 1). 

### 3.2. Molecular Identification

A total of 750,170 and 685,496 sequence reads were obtained for the samples collected from the Arboretum and the Dairy Barn traps, respectively. The number of post-filtered reads per site was 320,117 from the Arboretum and 291,847 from the Dairy Barn (Table 2). 

The highest number of barcode index numbers (BINs) (313) was recovered for the Arboretum, and only one BIN corresponded to a *Culicoides* species (Table 3). The Dairy Barn had 171 BINs identified corresponding to four separate *Culicoides* species. Three other BINs (TAX:701457, BOLD:AAA2294, and TAX:24896) from the Dairy Barn trap matched with species-level identification for cattle (*Bos taurus*), a known host for several species of *Culicoides*.

## 4. Discussion

A saturated salt (NaCl) solution provides many advantages when applied in a metabarcoding biosurveillance approach for flighted insects such as species of *Culicoides*. One such benefit is that a salt solution protocol has fewer transportation regulations, is non-toxic, is non-flammable, is easier to store, and is less costly than protocols using an alcohol-based collection solution [16,17]. Here, we demonstrate that *Culicoides* specimens remained morphologically intact in the salt trap solution overnight with no visible morphological interruptions or faults and remained useful for morphological identification methods (Figure 2 and Figure 3). In addition to the morphology of *Culicoides* remaining unaffected by the saturated salt solution, the use of a salt trap solution is shown to be a valid reservoir of eDNA. 

In the Dairy Barn trap, the molecular and morphological identifications were congruent for *C. biguttatus* (BOLD:AAG6468, HQ583003, and JF879904) and *C. stellifer* (BOLD:ABA0803 and KR659902) (Table 3), where eDNA was detected for these species. Two species, *C. crepuscularis* (n = 1) and *C. travisi* (n = 1), were identified morphologically but were not found using eDNA methods. eDNA was detected for *C. obsoletus* (BOLD:AAO7718 and MW642460) (Table 3), and, morphologically, the subgenus *Avaritia* was detected. *C. obsoletus* is in the subgenus *Avaritia*¸ showing that eDNA protocols may be able to identify specimens to a lower taxonomic level than morphological identification, provided there is an accurate DNA barcode reference library. Nevertheless, no eDNA detection from *Culicoides* occurred when there was a morphologically identified abundance lower than three specimens for the species. This lack of detection indicates that traps should remain for more extended periods at sites of lower densities of target taxa to maximize collections. It also suggests that a minimum amount of eDNA biomass from *Culicoides* may need to be shed into the solution to be detectable using the present protocol. Overall, these results are consistent with previous studies indicating a positive correlation between the abundance of specimens for a particular taxon and its detection probability [33]. Specific sensitivity tests, expanding the number of traps, and evaluating extended collection periods and different sampling storage temperatures are suggested to establish eDNA detection limits for target taxa.

Our results also indicate the need to test the stability of *Culicoides* eDNA and morphological integrity in saturated salt trap solutions for different periods in the presence and absence of soap. The only reason that the soap was included in the collection fluid was to act as a surfactant. The integrity of eDNA in saturated salt trap solutions has been tested for general arthropod detection, suggesting its stability for up to four weeks [16]. With positive detection of *Culicoides* DNA in the traps and previous research indicating the stability of eDNA in the high salt trap solutions of up to four weeks while also maintaining morphological characteristics, the method presented here is ideal for use in a regulatory capacity. Furthermore, the use of a solution with ingredients that are easily accessible and can be shipped with no dangerous goods concerns is ideal.

In the Arboretum trap, the molecular and morphological identifications did not match. Using molecular identifications, eDNA was detected from *C. mulrenanni* (BOLD:ACC8633) (Table 3), which was not one of the morphologically identified species of *Culicoides* from this trap. On BOLD, there are currently only two samples for this BIN, and both are from South Carolina. The quality of the species images in BOLD was not adequate to verify this species’ morphological identification when using the original species description [34]. Additionally, *C. mulrennnani* has not been identified to be in Ontario [10], but has been found in the state of New York, bordering Ontario to the south [35]. However, females of *C. mulrennani* are morphologically very similar to other species that are found in Ontario, such as *C. biguttatus* and *C. spinosus* [27,29], and all three of these species are placed in the same subgenus, *Silvaticulicoides* [36]. There are several possible explanations for the mismatch in the Arboretum trap: (1) the two samples on BOLD were misidentified as *C. mulrennani*; (2) our morphological identifications of *C. bigutattus* specimens were erroneous; (3) there is a regional difference in the morphology of *C. mulrennani* within its range that has not been documented, thus explaining why this species has never been identified in Ontario; or (4) the COI barcode for *Silvaticulicoides* is too similar species-wise to allow for differentiation, and perhaps this BIN encompasses multiple species within *Silvaticulicoides*. Regardless of the explanation, this mismatch emphasizes the need for a complete DNA barcode reference library with the applicable metadata, including detailed collection data, images, and molecular methods for each sample.

A limitation encountered in this study was the lack of images and morphological identification methods on the BOLD database for the BIN matches obtained in our eDNA samples. For all of the BINs in Table 3, the BOLD images were insufficient to verify the morphological identification of this reference library using existing taxonomic keys [9,22]. For *Culicoides* specimens with very distinctly patterned wings, such as *C. stellifer*, verifying the morphological identification would be more accurate and easier than *Culicoides* specimens with plain and/or similar-looking wings, such as *C. biguttatus* vs. *C. travisi*. This issue outlines the importance of having image metadata for other important morphological diagnostic characteristics for *Culicoides* samples, such as, but not limited to, clear images of the third palpal segments, antennomere ratios, leg segment patterns, and female internal genitalia. 

In the Dairy Barn trap, the amplification of *Bos taurus* (cattle) was observed. This molecular detection is justifiable given a number of the collected specimens displaying signs of recent bloodmeals (Figure 3). Although *Bos taurus* is outside our target taxa, its molecular identification along with the identified presence of recent bloodmeals in the collected *Culicoides* provides an excellent example for how this protocol could be expanded for future surveys to target both taxa and secondary taxa, be that in the form of bloodmeal [37], or other associated pathogens such as bacteria, fungi, or viruses. It also reinforces eDNA metabarcoding as a potentially powerful tool to analyze predator/prey relationships or pathogen/vector relationships.

## 5. Conclusions

The present study demonstrates that saturated salt (NaCl) solutions are a valid substitute for alcohol-based fluids for collecting *Culicoides* via conventional UV light traps. eDNA shed from specimens, and the morphological characteristics of the specimens themselves are preserved, allowing for species-level identifications using classical morphological identifications and eDNA metabarcoding techniques. Future studies should include the effect of different collection fluids (e.g., saturated salt solution, water with soap, and alcohol-based fluids such as ethylene/propylene-glycol) on specimens’ morphology and eDNA stability for extended incubation times at different temperatures. In addition, the success of eDNA metabarcoding identification relies on the content of molecular sequence libraries with morphologically identified specimens and associated molecular information and metadata. To further the efficacy of the methods presented here, there needs to be a continued effort to populate databases with high quality records of target taxa and closely related taxa to support metabarcoding efforts.

## Figures and Tables

**Table 1:** *Culicoides* taxonomic rank and abundance based on morphological identification.

Taxa	Arboretum	Dairy Barn
*Culicoides biguttatus*	3	13
*Culicoides crepuscularis*	-	1
*Culicoides stellifer*	-	13
*Culicoides travisi*	1	1
Subgenus *Avaritia*	3	7

**Table 2:** The number of reads after processing raw sequences, BINs, and number sequences associated with BIN identification, remaining OTU counts, percentage of filtered reads (Filtered %), and percentage of dereplicated reads (Dereplicated %) per sample.

Run Name	Reads	BINs	# Sequences	OTU Count	Filtered %	Dereplicated %
FAP3ST-ARB-04-COI	320,117	313	284,362	61	0.26	67.67
FAP3ST-DAB-03-COI	291,847	171	239,954	48	0.13	65.1

**Table 3:** *Culicoides* spp. identified per trap using the reference library for BINs found in the BOLD database (mBRAVE) and GeneBank database (Geneious). Included are the reference library’s BIN taxon identifier (BIN.Taxon.ID)/GenBank accession number (Accession), the taxonomic rankings of the species in the reference library (Order, Family, Genus, Species), the number of corresponding DNA sequences (Seq), average percent mean similarity (MS (%)), mean overlap (MO (bp)), percentage of identical sites (IS (%)), percentage of pairwise identity (PI (%)), and average bp length analyzed (Length).

**Arboretum (mBRAVE)**								
**BIN/Taxon ID**	**Order**	**Family**	**Genus**	**Species**	**Seq**	**MS (%)**	**MO (bp)**	**Length**
BOLD:ACC8633	Diptera	Ceratopogonidae	*Culicoides*	*Culicoides mulrennani*	3	98.06	395	395
**Dairy Barn (mBRAVE)**								
BOLD:AAG6468	Diptera	Ceratopogonidae	*Culicoides*	*Culicoides biguttatus*	103	99.45	397.6	397.47
BOLD:ABA0803	Diptera	Ceratopogonidae	*Culicoides*	*Culicoides stellifer*	4	97.64	402.5	402.5
BOLD:AAO7718	Diptera	Ceratopogonidae	*Culicoides*	*Culicoides obsoletus*	69	99.06	391.25	399.09
**Dairy Barn (Geneious)**								
**Accession**	**Order**	**Family**	**Genus**	**Species**	**Seq**	**IS (%)**	**PI (%)**	**Length**
HQ583003, JF879904	Diptera	Ceratopogonidae	*Culicoides*	*Culicoides biguttatus*	10	94.92	94.96	340.2
KR659902	Diptera	Ceratopogonidae	*Culicoides*	*Culicoides stellifer*	2	98	98	350
MW642460	Diptera	Ceratopogonidae	*Culicoides*	*Culicoides obsoletus*	2	100	100	351

## Data Availability

Raw data (FASTQ files) and specimen images available from the University of Guelph Research Data Repository (https://doi.org/10.5683/SP2/RMSBC8) and the Barcode of Life Data System (DS-CULS2021; dx.doi.org/10.5883/DS-CULS2021), respectively.

## Supplementary Materials

The following are available online at https://www.mdpi.com/article/10.3390/insects12050401/s1, Supplementary material S1: interactive version of the map (HTML) showing the collection sites, Table S1: list of the 38 known species of *Culicoides* reported in Ontario, Canada.
