# Supplementary material for: Validation of an Effective Protocol for Culicoides Latreille (Diptera: Ceratopogonidae) Detection Using eDNA Metabarcoding
Source: Insects. 2021 Apr 30;12(5):401. doi: 10.3390/insects12050401 (PMC8146839; doi:10.3390/insects12050401)
Supplement: Supplementary file 1 [file insects-12-00401-s001.zip › insects-1180709-supplementary/Table S1.pdf]

Species	Reference for the original description
C. alexanderi	Wirth and Hubert, 1962
C. arboricola	Root and Hoffman, 1937
C. atchleyi	Wirth and Blanton, 1969a
C. bickleyi	Wirth and Hubert, 1962
C. biguttatus	Coquillett, 1901
C. chiopterus	Meigen, 1930
C. cockerellii	Coquillett, 1901
C. crepuscularis	Malloch, 1915
C. denticulatus	Wirth and Hubert, 1962
C. downesi	Wirth and Hubert, 1962
C. flukei	Jones, 1956
C. footei	Wirth and Jones, 1956
C. franclemonti	Cochrane, 1974
C. frohnei	Wirth and Blanton, 1969b
C. furensoides	Williams, 1955
C. guttipennis	Coquillett, 1901
C. haematopotus	Malloch, 1915
C. jamnbacki	Wirth and Hubert, 1962
C. nanus	Root and Hoffman, 1937
C. obsoletus	Meigen, 1818
C. paraimpunctatus	Borkent, 1995
C. parapiliferus	Wirth and Blanton, 1974
C. piliferus	Root and Hoffman, 1937
C. sanguisuga	Coquillett, 1901
C. snowi	Wirth and Jones, 1956
C. sonorensis	Wirth and Jones, 1957
C. sphagnumensis	Williams, 1955
C. spinosus	Root and Hoffman, 1937
C. stellifer	Coquillett, 1901
C. stilobezzioides	Foote and Pratt, 1954
C. testudinalis	Wirth and Hubert, 1962
C. travisi	Vargas, 1949
C. utowana	Jamnback, 1965
C. variipennis	Coquillet, 1901
C. venustus	Hoffman, 1925
C. villosipennis	Root and Hoffman, 1937
C. wisconsinensis	Jones, 1956
C. yukonensis	Hoffman, 1925
